# Dataset on the reproductive period of three local species in a tropical sub-mountainous forest

**DOI:** 10.1016/j.dib.2019.104238

**Published:** 2019-07-06

**Authors:** Susana Paulina Dewi, Tati Suryati Syamsudin, Endah Sulistyawati

**Affiliations:** School of Life Sciences and Technology, Institut Teknologi Bandung (ITB), Indonesia

**Keywords:** *Castanopsis argentea*, *Saurauia microphylla*, *Schima wallichii*

## Abstract

This data article presents the reproductive period of three local species in a tropical sub-mountainous forest (1000–1300 m above sea level). The tree species were *Castanopsis argentea, Saurauia microphylla and Schima wallichii*. The reproductive periods were determined by the duration of flowering, flowering-fruiting, and fruiting of the tree species. Observation on the duration of the reproductive period was conducted by counting the number of flowering (flo), flowering-fruiting (flo-fru), and fruiting (fru) trees every month for 24 months successively from July 2015 to June 2017. Analyzed data is provided in Table 1. Primary data is presented in Supplementary Tables 1–3.

**Table:** Specifications table

Subject area	Agricultural and Biological Sciences
More specific subject area	Forestry
Type of data	Table, figure
How data was acquired	Observation on the existence of flowers, flowers and fruits, fruits
Data format	Primary and analyzed
Experimental factors	Number of flowering, flowering-fruiting, fruiting trees
Experimental features	Reproductive periods shown by the percentage of flowering, flowering-fruiting, and fruiting trees
Data source location	Conservation area of Mount Masigit-Kareumbi, Sumedang Regency, West Java, Indonesia (6° 51′ 31″–7° 00′ 12″ S latitude and 107° 50′ 30″–108° 1′ 30″ E longitude)
Data accessibility	The data is available within this article and accessible to the public
Related research article	Unpublished data

**Table:** 

**Value of the data** • This data informs the duration of the reproductive period (flowering, flowering-fruiting, fruiting) of *Castanopsis argentea*, *Saurauia microphylla*, and *Schima wallichii*, • The data could be used by other researchers on forest restoration. • This data could support seed production for forest restoration.

## 1 Data

**Fig. 1:**
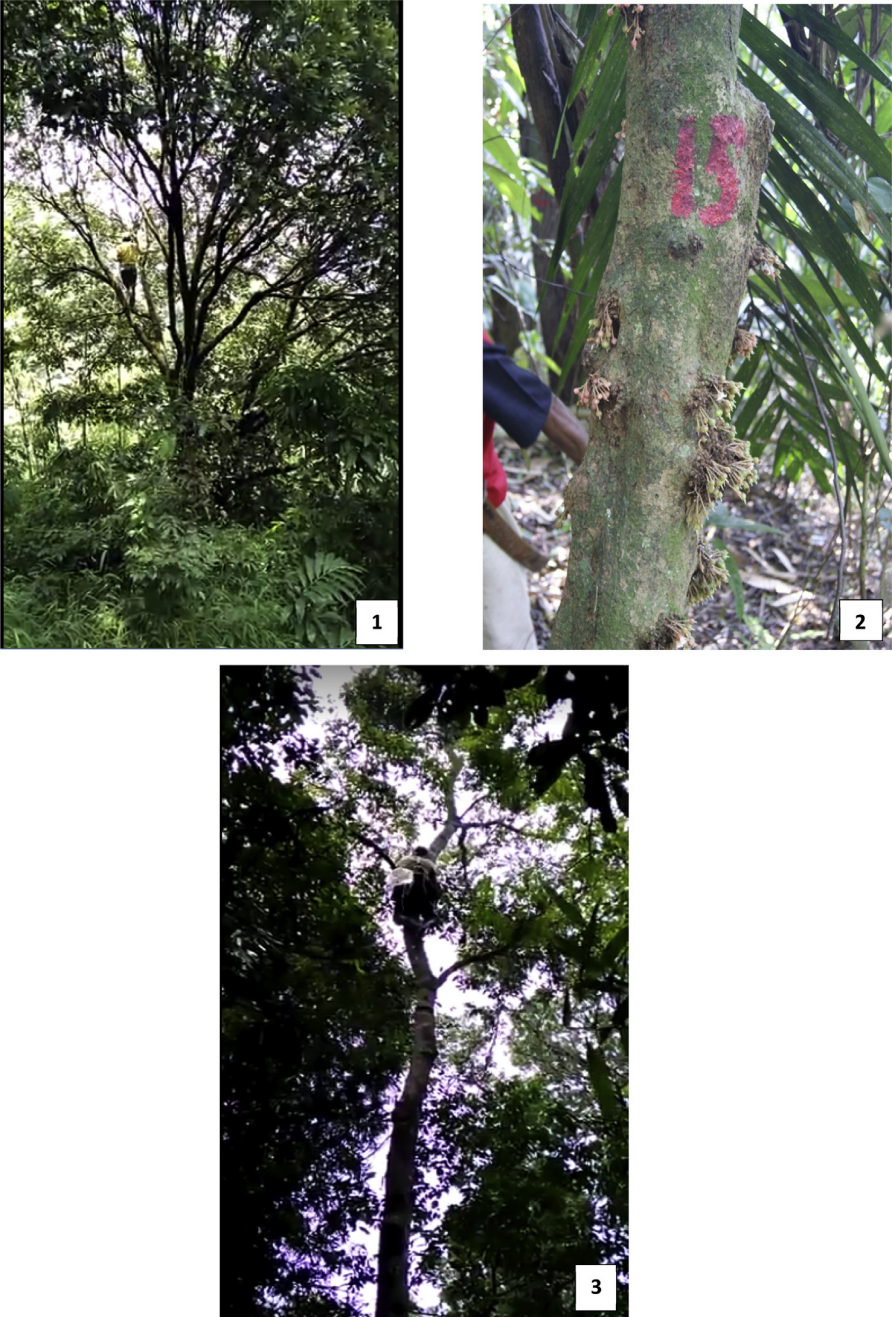
Local tree species: 1 = *Castanopsis argentea*; 2 = *Saurauia microphylla*; 3 = *Schima wallichi*.

Table 1 contains the analyzed data on the percentage of individuals flowering (flo), flowering-fruiting (flo-fru), and fruitings (fru) of three local species: *Castanopsis argentea*, *Saurauia microphylla*, and *Schima wallichii (*Fig. 1). The flowering phase determined by the presence of flowers buds until anthesis of flowers. The fruiting phase determined by the presence of mixture immature and mature fruits. Meanwhile, the flowering-fruiting phase determined by the presence of flowers and fruits in the same time. Primary data is presented in Supplementary Tables 1–3.

**Table 1:** The reproductive phases of three local species (%).

Observation time (month)	Flower			Flower-Fruit			Fruit		
	Sp.1	Sp.2	Sp.3	Sp.1	Sp.2	Sp.3	Sp.1	Sp.2	Sp.3
July 2015	0.0	37.5	11.8	0.0	50.0	0.0	0.0	0.0	0.0
August 2015	6.7	37.5	8.8	0.0	0.0	11.8	40.0	12.5	8.8
September 2015	20.0	50.0	2.9	0.0	12.5	0.0	6.7	12.5	38.2
October 2015	33.3	50.0	0.0	0.0	0.0	0.0	0.0	12.5	55.9
November 2015	0.0	50.0	0.0	6.7	0.0	5.9	20.0	12.5	55.9
December 2015	6.7	37.5	23.5	0.0	37.5	0.0	26.7	25.0	20.6
January 2016	13.3	25.0	2.9	0.0	62.5	8.8	26.7	12.5	14.7
February 2016	20.0	12.5	23.5	0.0	50.0	0.0	20.0	25.0	2.9
March 2016	0.0	12.5	5.9	0.0	0.0	2.9	0.0	62.5	20.6
April 2016	20.0	12.5	0.0	0.0	50.0	0.0	0.0	12.5	20.6
May 2016	0.0	62.5	0.0	0.0	0.0	0.0	0.0	0.0	20.6
June 2016	0.0	100.0	0.0	0.0	0.0	0.0	0.0	0.0	20.6
July 2016	0.0	0.0	14.7	0.0	0.0	0.0	0.0	62.5	0.0
August 2016	0.0	12.5	29.4	0.0	0.0	0.0	0.0	50.0	0.0
September 2016	13.3	37.5	14.7	0.0	12.5	5.9	0.0	25.0	11.8
October 2016	0.0	62.5	0.0	0.0	0.0	0.0	0.0	12.5	20.6
November 2016	6.7	75.0	2.9	0.0	12.5	0.0	0.0	0.0	2.9
December 2017	6.7	37.5	20.6	0.0	25.0	0.0	0.0	37.5	0.0
January 2017	0.0	12.5	23.5	6.7	50.0	0.0	0.0	12.5	2.9
February 2017	6.7	62.5	0.0	0.0	12.5	0.0	0.0	12.5	17.6
March 2017	0.0	25.0	0.0	0.0	37.5	0.0	6.7	12.5	5.9
April 2017	0.0	37.5	8.8	0.0	12.5	0.0	6.7	12.5	2.9
May 2017	0.0	37.5	5.9	0.0	25.0	0.0	6.7	12.5	5.9
June 2017	6.7	37.5	14.7	0.0	25.0	0.0	6.7	0.0	0.0

Note: Sp.1 = *Castanopsis argentea*; Sp.2 = *Saurauia microphylla*; Sp.3 = *Schima wallichii*.

## 2 Experimental design, materials, and methods

The study was conducted in a tropical sub-mountainous forest (about 1000–1300 m above sea level). Mean annual rainfall is 1900 mm per year with relative humidity 60%–90% and temperature average is 23 °C. Mostly the study sites are covered by natural forests and topographic condition is rather steep.

The choice of these three species is based on its dominance, it's a native species, have clear reproductive phases and it owned fruit that animals preferred. *Saurauia microphylla* has other uniqueness compare to *Schima wallichii* and *Castanopsis argentea*. It is an endemic flora of Java Island and listed under the IUCN Red List of Threatened Species 2016 (World Conservation Monitoring Center 1998). Furthermore, these three species will be designated as a seed source for forest restoration activities.

A total of twenty plots were set across all study sites purposively. The arrangement of plot in the field was modified from Ref. [1]. Observation plot (20 m × 20 m) was set every five plots in every 100 m interval of altitude. The plots had to be covered by natural vegetation (not planted), accessible, have at least five species of trees present inside the plot, and should not be prone to landslides. Individual trees in the observation plots were selected based on their diameter at breast height (DBH). Trees with a DBH of more than 10cm were assumed to have entered the reproductive phase. Each selected tree was labeled with a unique number so that it could be identified. At each plot, the reproductive phases (consisting of flowering (flo), flowering-fruiting (flo-fru), and fruiting (fru)) were observed following [2], [3] with some modifications. The observation of flowering (flo) and fruiting (fru) phases was carried out on the duration (days) of the first flower or fruit formation to the last amongst its individuals. While the flowering-fruiting (flo-fru) phase was observed by the duration of the flower and fruit presence in the same time. The observations were conducted every month simultaneously from July 2015 to June 2017. The percentage of trees in a particular phenophase of each species observed is a proportion of the total number of selected trees found in all observation plots.

## Appendix A Supplementary data

Multimedia componenet 1 Multimedia componenet 1

Multimedia componenet 2 Multimedia componenet 2

Multimedia componenet 3 Multimedia componenet 3

## References

[1] Sutherland W.J. (2006). Ecological Census Techniques: a Handbook. https://epdf.tips/ecological-census-techniques-a-handbook.html.

[2] Sellamuthu S., Lalitha V. (2010). Plant diversity and phenological pattern in the montane wet temperate forests of the southern western Ghats, India. For. Stud. China.

[3] Singh K.P., Kushwaha C.P. (2006). Diversity of flowering and fruiting phenology of trees in a tropical deciduous forest in India. Ann. Bot..

